# Clinical research progress on intrathecal glucocorticoids in the treatment of infections: A review

**DOI:** 10.1097/MD.0000000000038123

**Published:** 2024-05-17

**Authors:** Zixv Lv, Yingying Liu

**Affiliations:** a Department of Pain, Zibo First Hospital, Zibo, Shandong, China; b Department of Neurosurgery, Zibo First Hospital, Zibo, Shandong, China.

**Keywords:** central nervous system diseases, infections, intrathecal glucocorticoids, reviewed

## Abstract

In some infectious diseases, pathogenic microorganisms can directly or indirectly cause significant inflammatory reactions in the central nervous system, leading to severe neurological dysfunction, such as suppurative meningitis, tuberculous meningitis, and febrile infections. related epilepsy syndrome, etc. In these diseases, adjuvant administration of glucocorticoids is necessary to inhibit the release of proinflammatory cytokines, and intrathecal administration can deliver the drug more directly to the target. In this article, the authors studied intrathecal glucocorticoids for the treatment of infectious inflammatory reactions in terms of pharmacological effects and mechanisms, pharmacokinetics, clinical application, and safety. The authors concluded that the article could help provide new treatment strategies for infectious diseases.

## 1. Introduction

Infections can directly or indirectly cause nervous system dysfunction. Acute and chronic inflammatory diseases caused by pathogenic microorganisms such as bacteria, fungi, and viruses that invade the parenchyma, capsule, and blood vessels of the central nervous system (CNS) are called infectious CNS diseases. In some CNS diseases, although pathogenic microorganisms have not invaded the CNS, they are considered to be related to the immune response or toxins produced by the infection, which are called infection-related CNS diseases, such as febrile infection-related epilepsy syndrome. In the current treatment of CNS dysfunction caused by infection, adjuvant glucocorticoid therapy can significantly improve clinical outcomes, including reducing mortality, which may be related to the fact that glucocorticoids can improve the inflammatory response caused directly or indirectly by infection.^[1]^ Intrathecal medication is a method of directly injecting drugs into the subarachnoid space, so that the drugs can directly act on the brain and spinal cord through cerebrospinal fluid circulation.

This route of administration is not restricted by the blood–brain barrier (BBB) and acts accurately and rapidly. It is an important way to treat diseases related to the CNS. This article will discuss the use of intrathecal glucocorticoids (IT-GC), review the existing data on intrathecal steroid use in infectious and infection-related CNS diseases.

## 2. Mechanism of action of IT-GC

Intrathecal drugs act directly on the pial membrane and central nervous. Glucocorticoids have powerful anti-inflammatory effects and can inhibit inflammatory reactions caused by pathogenic organisms, immunity and other factors. The main mechanism is to inhibit the expression of cyclooxygenase and the production of cytokines such as interleukin-1 and tumor necrosis factor (TNF).

The pia mater is rich in blood vessels. In the early stage of acute inflammation, intrathecal injection of glucocorticoids can increase intrapial vascular tone, reduce congestion, reduce vascular permeability, inhibit white blood cells, reduce the release of various inflammatory factors, and reduce local nervous system tissue exudation, edema, thereby improving neurological symptoms such as headache, meningeal irritation, cerebral edema, and epilepsy. In addition, glucocorticoids can act on the BBB to improve nutrient uptake, waste removal, and enzymatic processes, which will facilitate the elimination of inflammation.^[2,3]^

Microglia are macrophage-like cells that reside in the CNS and when activated with appropriate antigens, or by pathological states such as necrotic and apoptotic cells, they produce pro-inflammatory cytokines such as interleukin-1, TNF, and chemokines. These cytokines are produced in defense of the organism, but when they are activated, they produce highly inflammatory products with effects that are deleterious for neurons and for the life of the individual. Excessive production of cytokines and chemokines by microglia activation can lead to neurological diseases.^[4–6]^ Intrathecal glucocorticoids inhibit the production of these inflammatory mediators.

It should be noted that inflammatory response is a defensive mechanism of the body, so glucocorticoids can also cause the spread of infection while suppressing inflammation and reducing symptoms. Commonly used intrathecal steroid hormones include hydrocortisone, prednisolone, triamcinolone, and dexamethasone. It should be noted that the oxygen on the 11th carbon atom of cortisone and prednisone needs to be converted into hydroxyl groups in the liver to produce hydrocortisone and prednisolone to be active, so they should not be administered intrathecally.^[7]^

## 3. Pharmacokinetics of IT-GC

The BBB isolates the cerebrospinal fluid from the blood flow. Except for water, soluble gases and fat-soluble small molecules (400–600 Da), other organic molecules cannot freely pass through the endothelium.^[8]^ The subarachnoid space therefore forms a relatively independent space, and when glucocorticoids are injected intrathecally, the drug first diffuses freely in the cerebrospinal fluid. Drug concentrations in brain parenchymal interstitial fluid and cerebrospinal fluid are considered pharmacologically similar because there is no limiting barrier.^[9,10]^ In addition, BBB permeability is bidirectional, and intrathecal exogenous steroids can diffuse into the peripheral circulation system through the BBB, which is related to the bidirectional transport function of P-glycoprotein transporters belonging to the family of ATP-binding cassette transporters.^[11,12]^ When there is moderate BBB dysfunction, there are fewer active transporters and the diffusion of intrathecally injected exogenous glucocorticoids into the circulation is reduced, resulting in higher concentrations in the cerebrospinal fluid.

### 3.1. Clinical application of IT-GC

#### 3.1.1. Bacterial infectious diseases

Bacterial infectious diseases of the CNS may be spontaneous or may be complications after trauma or surgery, which significantly affects the patient’s prognosis. Severe infections may even lead to disability or death.^[13]^ First-line treatment is prompt empiric intravenous antibiotics and adjuvant steroids, but should not be used if Listeria monocytogenes infection is confirmed.^[14]^ When the condition is critical or when systemic antibiotics are not effective, intrathecal administration of antibiotics combined with steroids can be considered to achieve better clinical results.^[15]^

#### 3.1.2. Suppurative meningitis

Suppurative meningitis is an acute inflammatory response syndrome caused by purulent bacteria that invade the leptomeninges. It is usually called acute bacterial meningitis. It is mainly characterized by fever, increased intracranial pressure, meningeal irritation, and purulent changes in cerebrospinal fluid. Clinical characteristics, the mortality rate is 10%, and the incidence of sequelae such as hearing loss, mental retardation, and quadriplegia after treatment is as high as 30%.^[16,17]^ Brain tissue damage in patients with purulent meningitis is not only due to direct attack by bacteria, but is also closely related to the host’s immune response to certain components released by bacteria. Cytokines are released, leading to further damage to the BBB, leading to neuron death.^[18]^ In addition, this inflammatory response will activate the complement system and coagulation cascade, promote thrombosis, lead to cerebrovascular disease and hypercoagulation, and lead to cerebral ischemia.^[19]^

Corticosteroids can reduce intrathecal inflammatory reactions and significantly reduce neurological sequelae such as hearing damage, epilepsy, and ataxia, but they cannot reduce mortality.^[20]^ The timing of application of steroid corticosteroids should be before or at the same time as the first dose of antibiotic treatment to prevent inflammatory reactions caused by antibiotic bacteriolysis, but the antibiotic must be able to kill the meningitis bacteria. If the presumed cause of infection is incorrect and the antibiotic cannot kill organism, use of corticosteroids may result in worse outcomes.^[21,22]^ Most studies used intravenous dexamethasone at a dose of 0.15 mg/kg every 6 hours for 4 days.^[23]^ There are some clinical reports of intrathecal dexamethasone (IT-DEX) in China, but the necessity remains to be discussed. In a prospective study of 90 children with refractory purulent meningitis, Ma Xiaoyun et al, the control group was treated with intravenous meropenem alone, and the observation group was treated with intrathecal vancomycin and dexamethasone in addition to the control group. The dose of dexamethasone is 2 to 3 mg/time, injected once every 1 to 2 days, and the course of treatment is 1 week. After 7 days of treatment, the levels of inflammatory markers (TNF-α, CRP, PCT) in the observation group decreased more significantly, and the improvement rate of symptoms and signs was higher, but there was no significant difference in neurological sequelae such as hearing impairment.^[24]^

#### 3.1.3. Tuberculous meningitis (TBM)

TBM is a diffuse nonsuppurative inflammatory disease caused by *Mycobacterium tuberculosis* invading the subarachnoid space. In addition to the leptomeninges and brain arachnoid, it can also invade the brain parenchyma and cerebral blood vessels. The onset is insidious, with symptoms of tuberculosis poisoning, meningeal irritation, increased intracranial pressure, brain parenchymal damage, and cranial nerve damage as the main manifestations, resulting in death or disability in almost half of the patients.^[25]^ Most tissue damage can be attributed to a dysregulated host inflammatory response rather than direct invasion of *Mycobacterium tuberculosis*; in fact, the bacterial load in the cerebrospinal fluid of most individuals is very low.^[26,27]^ During the course of the disease, tuberculous exudates cause subarachnoid adhesion and atresia, or obstruction of the cerebrospinal fluid circulation pathway due to occlusion of the middle and lateral foramen of the fourth ventricle, secondary to ventricular enlargement and hydrocephalus. Inflammatory exudates invade the brain parenchyma and directly cause cerebral edema. In addition, inflammatory exudates can also cause adhesion of the facial nerve, abducens nerve and posterior cranial nerve.^[28]^

Intracerebral inflammation has been considered an important determinant of prognosis in TBM. Adjuvant glucocorticoid therapy can reduce inflammation and thereby improve the prognosis of TBM, especially mortality.^[29,30]^ Intrathecal glucocorticoid injection can be given at the same time as systemic drug therapy to improve the efficacy. Nobuyuki Ashizawa^[31]^ reported a case of TBM refractory to intrathecal injection of isoniazid and steroids. The patient was a 52-year-old woman. She still had fever, headache and vomiting despite systemic antituberculosis and steroid treatment. Brain MRI showed meningitis. The disease worsened. After strengthening the treatment of meningitis by placing a lumbar drainage tube and administering isoniazid 100 mg and steroids (prednisolone 15–20 mg or dexamethasone 1.65 mg) intrathecally through intermittent puncture, the fever subsided quickly, and the headache and nausea were relieved. Systemic steroids were subsequently tapered, and eventually the patient was continued on isoniazid and rifampicin without recurrence of meningitis. S. P. Khatua^[32]^ et al conducted a prospective study of pediatric TBM patients, in which 43 patients under 7 years of age received oral or intrathecal steroids in addition to all antituberculosis treatments. The 20 patients in the oral group took 10 to 20 mg of prednisolone 3 to 4 times a day for 3 weeks and then gradually reduced it until it was completely stopped. The remaining 23 patients received intrathecal hydrocortisone 12.5 to 25 mg for 3 weeks, initially once a day for 6 consecutive injections, and then once every other day for a further 6 injections. In patients treated with intrathecal hydrocortisone, survival rates were much higher, disease duration was much shorter, coma and hospital stay were much shorter, and sequelae were much less severe. Li K^[33]^ et al conducted a retrospective study on adult patients. Patients in Group A received only systemic antituberculosis treatment, while patients in Group B received twice-weekly intrathecal isoniazid (50 mg) and prednisolone (25 mg) in addition to systemic antituberculosis treatment.198 patients with TBM were included in a total of 60 cases for comparison through propensity score matching, with 30 cases in each group. The study found that adding IT INH to systemic antituberculosis treatment and prednisolone may be effective in improving outcomes in adult patients with TBM. Therefore, large-scale, prospective, and randomized controlled trials are necessary to find the optimal timing and indications for IT treatment.

### 3.2. Febrile infection-related epilepsy syndrome (FIRES)

FIRES is a rare and catastrophic epileptic encephalopathy.^[34]^ FIRES occurs in all ages, but usually occurs in children 4 to 9 years old. There are no clear differences between childhood and adult-onset syndromes.^[35]^ A history of infection is a prerequisite for FIRES, which has fever symptoms 2 weeks to 24 hours before onset, with or without fever. The pathogenesis of FIRES is related to the fulminant inflammatory response in the CNS. Infection triggers rapid and transient induction of specific inflammatory molecules in the brain, intrathecal overproduction of pro-inflammatory cytokines and chemokines, some of which have proconvulsant activity, promoting epileptic seizures, which contribute to the maintenance of the inflammatory state, forming A vicious cycle leads to status epilepticus.^[36]^ FIRES is a type of new-onset refractory status epilepticus. Usually, the epilepsy cannot be stopped even after treatment with 2 or more drugs.^[34,37]^

Although there are currently no specific effective treatments for FIRES other than the ketogenic diet, given the causal role of inflammation in FIRES, steroid modulation of the immune system could be attempted.^[38]^ IT-DEX has been used. Horino A^[39]^ et al performed intrathecal dexamethasone treatment on 6 FIRES patients after completing at least one course of conventional immunotherapy. The starting dose of IT-DEX was 0.15 to 0.25 mg/kg/day. The treatment is repeated 4 times, with additional times depending on the patient’s specific condition. The dosing interval is 1 to 6 days. Seizure spread and EEG background activity improved after IT-DEX in all patients, narcotics were discontinued after a median of 5.5 days, mechanical ventilation was discontinued after a median of 26.5 days, and the duration of critical disease stages was significantly shortened without adverse events. occur. Mehta NP^[40]^ after 6 times IT-DEX in a 9-year-old male pediatric patient dependent on continuous anesthetic infusion the seizures ceased, the anesthetic infusion was stopped quickly, and inflammatory markers improved. At the time of discharge, he was walking with assistance, bilingual, and taking food by mouth. Tetsuya Ioku^[41]^ treated a 16-year-old male patient with 2 courses of intrathecal dexamethasone injection (6.6 mg/time, once every other day, 7 times/course) in addition to other antiepileptic treatments. The patient was on the 170th day, he was weaned off the ventilator and able to live at home, but some sequelae remained.

## 4. Safety of IT-GC

### 4.1. Neurotoxicity of IT-GC

There are currently no reports on the toxicity of intrathecal steroids to the human CNS, but excessive doses of intrathecal steroids per unit time may cause inflammation of the nervous system.^[42]^ J S Kroin^[43]^ found in an in vivo rat study that low-dose continuous intrathecal infusion of dexamethasone was safe, but that higher doses may lead to increased inflammation. Schlatter J^[44]^ searched the relevant literature on intrathecal injection of methylprednisolone from 1940 to June 2016. Intrathecal injection of methylprednisolone can cause arachnoiditis, bladder dysfunction, headache, meningitis and other adverse reactions, but it is related to the additives of the preparation. related. Clinically, if a patient experiences adverse reactions due to corticosteroid treatment, it should be important to consider that the excipients of the preparation may be the potential cause of the problem.

### 4.2. Effects of IT-GC on plasma cortisol levels and adrenal function

IT-GS have a significant inhibitory effect on the hypothalamic–pituitary–adrenocortical axis, leading to adrenocortical insufficiency.^[45,46]^ Plasma cortisol will decrease temporarily after a smaller number of intrathecal injections and return to normal in more than 1 month.^[47]^ However, if intrathecal injection is repeated, the serum cortisol level will decrease more significantly, even to an almost undetectable level. In addition, corticosterone and estrogen will also be significantly reduced, which may cause irreversible damage to adrenocortical function.^[48]^ Therefore, adrenal function in these patients should be carefully monitored during intrathecal glucocorticoid injection therapy.

## 5. Other instructions

In addition to the above-mentioned bacterial meningitis, TBM, and FIRES, glucocorticoids can also be used in other CNS diseases caused by infection and dominated by excessive inflammatory reactions, but intrathecal injection is not required. Intrathecal injection is generally used in refractory diseases with more dangerous conditions, higher mortality, and obvious sequelae. For example, viral meningitis mostly has a benign course and can be treated conventionally without intrathecal injection. If the BBB is not invaded and its function is acceptable, the entry of glucocorticoids into the cerebrospinal fluid is not affected, and conventional application methods can be used. For example, herpes simplex virus encephalitis has an acute onset and serious damage to the CNS. Glucocorticoid treatment should be taken during the optimal window period. However, the main damage is to the brain parenchyma and does not invade the meninges. Glucocorticoids penetrate through The BBB is not affected and intrathecal injection is not necessary.^[49]^

The intrathecal administration methods of glucocorticoids are diverse, including lumbar puncture administration, translumbar indwelling catheter administration, transventricular catheter administration, etc., which can be selected according to the patient’s condition. For example, low-frequency administration can be selected. Each time a lumbar puncture is performed, the drug can be administered after a catheter is left in the lumbar subarachnoid space for continuous administration.^[50]^ When the patient has an indwelling ventricular catheter after surgery, the drug can be administered through the ventricular catheter. If the patient’s condition requires the cerebrospinal fluid to be sent for examination, 3 to 4 mL should be slowly released before intrathecal administration of the drug and then the drug should be injected. When injecting steroids, the cerebrospinal fluid should be withdrawn 5 to 6 times while injecting, which will help the distribution of the drug. After the injection is completed, the patient should lie on his back for 4 to 6 hours.

## 6. Summary

Intrathecal injection of glucocorticoids is a direct and effective way to directly inhibit the inflammatory response of the CNS caused by infection. It has been used in the treatment of bacterial meningitis, TBM, and FIRES. Although there are few reports on their application, existing evidence supports that IT-GC are more beneficial in bacterial meningitis, TBM, and FIRES. However, there are few reports on the application timing, optimal glucocorticoid type, and optimal dosage of medication, and future research needs to be further explored.

## Author contributions

**Writing – original draft:** Zixv Lv.

**Writing – review & editing:** Yingying Liu.
